# Validating the DIVERT scales, CARS, and EARLI for predicting emergency department visits in home health care in Japan: A retrospective cohort study

**DOI:** 10.1002/jgf2.738

**Published:** 2024-11-20

**Authors:** Takao Ono, Hiroko Watase, Takuma Ishihara, Taketo Watase, Kiho Kang, Mitsunaga Iwata

**Affiliations:** ^1^ Department of Emergency Medicine and General Internal Medicine Fujita Health University School of Medicine Toyoake Aichi Japan; ^2^ Midori Homon Clinic Nagoya Aichi Japan; ^3^ Innovative and Clinical Research Promotion Center Gifu University Hospital Gifu Japan

**Keywords:** CARS, DIVERT scale, EARLI, emergency department visits, home health care

## Abstract

**Background:**

The Detection of Indicators and Vulnerabilities for Emergency Room Trips (DIVERT) scale, the Community Assessment Risk Screen (CARS), and the Emergency Admission Risk Likelihood Index (EARLI) are scales that assess the risk of emergency department (ED) visits among home health care patients. This study validated these scales and explored factors that could improve their predictive accuracy among Japanese home health care patients.

**Methods:**

This was a single‐center retrospective cohort study. The primary outcome of unplanned ED visits was used to assess the validity of the DIVERT scale, CARS, and EARLI. Additionally, we examined whether the addition of patient age and receipt of advance care planning as variables on these assessments could enhance their precision.

**Results:**

Altogether, 40 (17.8%) had at least one ED visit during the 6 months study period. In these patients, the DIVERT scale, CARS, and EARLI of the patients with ≥1 ED visit was superior compared with no ED visit (both *p* < 0.05). The area under the curve (AUC) of the DIVERT scale, CARS, and EARLI were 0.62, 0.59, and 0.60, respectively. Adding patient age and receipt of advance care planning improved the AUC in all three scales.

**Conclusions:**

Our findings suggest that these assessment scales could be applicable to home health care patients in Japan. Furthermore, adding age and receipt of advance care planning as variables was found to enhance the predictive accuracy of the scales.

## Graphical abstract

This study validated the DIVERT scales, CARS, and EARLI for predicting emergency department visits among Japanese home health care patients. The area under the curve (AUC) of the DIVERT scale, CARS, and EARLI were 0.62, 0.59, and 0.60, respectively. We found that adding patient age and receipt of advance care planning improved the AUC in all three scales.

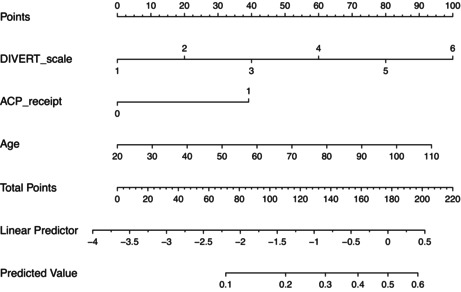

## 1 INTRODUCTION

Japan has an aging population,^1^ and between 14.8% and 47.4% of the population wish to spend the last phase of their life at home.^2^ With the rising proportion of older people, the cost of health care has also increased, creating substantial economic burdens on the health care system. Home health care has been shown to be less expensive than hospital admission,^3^ leading to an increase in both the number of patients who receive home health care and the number of home health care physicians. These figures are expected to continue rising until the late 2030s.^4^

Most home health care patients are older adults who can require emergency transport to emergency departments (ED).^5^ Emergency ED visits and hospitalization among older adults can be associated with the difficulty of continued living at home.^6^ Emergency ED visits and unexpected hospital admissions often disrupt their ability to live comfortably at home.^7^ Therefore, it is critical to ensure cooperation from the local community, including home health care services and the ED, to ensure their medical needs are met.^8^

To predict ED visits by home health care patients, different risk assessment scales have been developed: the Detection of Indicators and Vulnerabilities for Emergency Room Trips (DIVERT) scale, the Community Assessment Risk Screen (CARS), and the Emergency Admission Risk Likelihood Index (EARLI).^9^, ^10^, ^11^ The DIVERT scale was derived from the population‐level Resident Assessment Instrument Home Care (RAI‐HC) records,^12^ whereas CARS and EARLI were derived from patient questionnaires.^10^, ^11^ Their validity has been evaluated in six European countries^13^ but not in Japan.

To address this gap in the literature, we assessed the validity and predictive accuracy of the DIVERT scale, the CARS, and the EARLI among Japanese patients. We also investigated the extent to which the addition of age^14^ and receipt of advance care planning (ACP) to the scales could improve their predictive accuracy. Both of these factors have previously been found to be associated with ED visits.^14^, ^15^, ^16^ We evaluated the impact of these factors on the area under the curve (AUC) for the development of a Japan‐specific risk scale for ED visits.

## 2 METHODS

### 2.1 Study design and settings

This single‐center retrospective cohort study was approved by the Institutional Review Board of Fujita Health University Hospital and Midori Homon Clinic (approval number HM22‐368). To evaluate the risk of ED visits in home health care patients, including emergency transport and walk‐in visits, we selected the Midori Homon Clinic in Nagoya City in the Aichi prefecture (the fourth biggest prefecture in Japan, with a population of 7.553 million) that receives an average of 250 patients annually. The study period was from January 1 to June 30, 2022. All study participants provided written informed consent and the study was conducted in accordance with the tenets of the 2013 revision of the Declaration of Helsinki.

### 2.2 Participants

All patients who lived in their own homes and received home health care from this clinic for over 2 months during the study period were included. Those who were hospitalized or lived in nursing homes on January 1, 2022, were excluded from the study.

### 2.3 Data collection

The codes used in insurance claim processing were used to identify age, duration since the first home health care visit, and significant diseases and comorbidities.^17^ Factors that we believed clinically important, including age, socioeconomic status, total number of medications, duration since the first home health care visit, the presence of a caregiver, and receipt of ACP were confirmed with patients and their caregivers. ACP is a process of discussing end‐of‐life care to ensure the patients' preferences for care,^18^ especially regarding the most important aspects to the individuals, medical conditions they would want treatment for, and treatment they would want.^19^ It was conducted with patients, with or without their caregivers, initiated by a physician, and repeated whenever necessary. Detailed information on variables and data sources is provided in Table S1. All information was recorded using REDCap electronic data capture tools at Fujita Health University and anonymized to protect patient privacy.^20^

### 2.4 Outcome measurement

The outcome measure of interest was ED visits in the 6 months between January 1 and June 30, 2022. These included both ambulance and walk‐in arrivals.

### 2.5 Statistical analysis

The patients' characteristics were described using the mean ± standard deviation (SD) for continuous variables and number and percentage (%) for categorical variables. A two‐sided *p*‐value of <0.05 was considered statistically significant. In this study, we used univariate analysis to verify the improvement in accuracy provided by each factor because of the limited sample size. All analyses were performed using R statistical software v. 4.2.0 (R Foundation for Statistical Computing).

## 3 RESULTS

A total of 228 eligible patients were recruited by January 1, 2022, four of whom were excluded because they did not consent to participation. Of the remaining 224 patients, 40 (17.8%) had made at least one ED visit during the study period.

The mean age of the patients with ≥1 ED visit (*n* = 40) was 83.1 ± 12.3 years and that of the patients with no ED visits (*n* = 184) was 78.4 ± 16.6 years. There was no significant difference between the ages of the two groups (*p* = 0.09). Only five patients visited the ED multiple times. Approximately 45.0% of the patients with ≥1 ED visit and 54.3% of the patients with no ED visits were male (*p* > 0.99). The DIVERT scale of the patients with ≥1 ED and no ED visits were 2.5 ± 1.4 and 1.9 ± 1.0 (*p* = 0.001), respectively. The CARS of the two groups were 4.5 ± 2.8 and 3.6 ± 2.3, respectively (*p* = 0.05); and the EARLI were 13.2 ± 5.2 and 11.0 ± 5.0 (*p* = 0.02), respectively. The clinical characteristics of the 224 patients are shown in Table 1.

**TABLE 1:** Patient Characteristics in Home Care Patients With and Without Recent ED Visits.

	Mean ± SD or n (%)		
	All (*N* = 224)	≧1 ED visits (*n* = 40)	No ED visits (*n* = 184)
Age, years	79.2 ± 16.0	83.1 ± 12.3	78.3 ± 16.6
Gender, male	122 (54.5)	18 (45.0)	84 (45.7)
DIVERT scale	2.0 ± 1.1	2.5 ± 1.4	1.9 ± 1.0
CARS	3.8 ± 2.4	4.5 ± 2.8	3.6 ± 2.3
EARLI	11.4 ± 5.1	13.2 ± 5.2	11.0 ± 5.0
Duration since the first visit, months	30.0 ± 26.8	25.9 ± 25.6	31.0 ± 27.1
Number of medications	7.1 ± 4.2	6.9 ± 3.9	7.1 ± 4.2
Prior ED visits within 6 months	22 (9.8)	9 (22.5)	13 (7.0)
Receipt of ACP	85 (37.9)	22 (55.0)	63 (34.2)
Presence of the caregiver	190 (84.8)	36 (90.0)	154 (83.7)
Weight loss	34 (15.2)	12 (30.0)	22 (12.0)
Use of a urinary catheter	33 (14.7)	12 (30.0)	21 (11.4)
Medical history			
Urinary tract infection	11 (4.9)	4 (10.0)	7 (3.8)
Heart failure	119 (53.1)	26 (65.0)	93 (50.5)
COPD	12 (5.4)	2 (5.0)	10 (5.4)
Renal failure	18 (8.0)	4 (10.0)	14 (7.6)
Pneumonia	9 (4.0)	2 (5.0)	7 (3.8)
Stroke	70 (31.2)	9 (22.5)	61 (33.2)
Diabetes	70 (31.2)	12 (30.0)	58 (31.5)
Coronary artery disease	9 (4.0)	3 (7.5)	6 (3.3)

Abbreviations: ACP, advance care planning; COPD, chronic obstructive pulmonary disease; ED, emergency department; SD, standard deviation.

**FIGURE 1:**
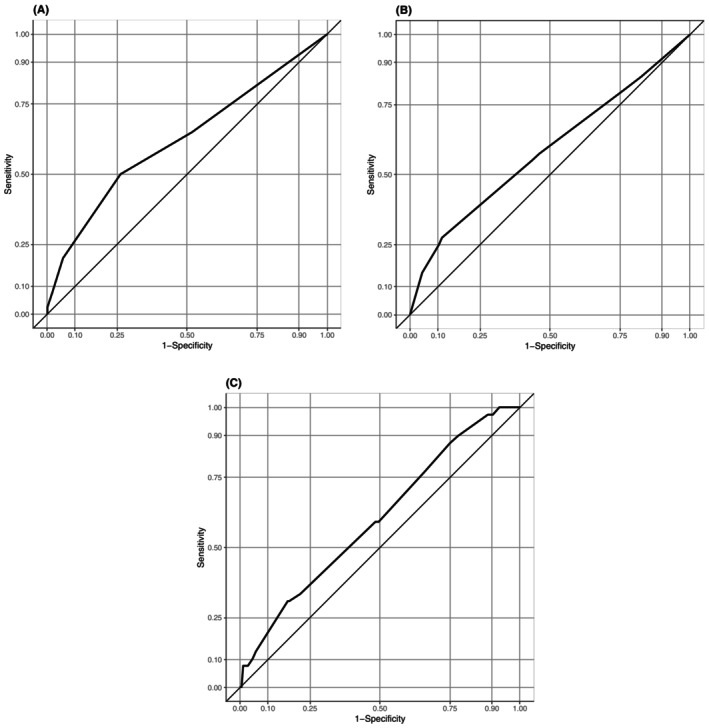
Receiver operating characteristic (ROC) curve analyses for each assessment scale: (A) the DIVERT scale, (B) the CARS, and (C) the EARLI. These ROC curves assessed the predictive performance of each assessment scale.

Univariate analyses found that the patients with ≥1 ED visit were more likely to be in receipt of ACP (odds ratio [OR], 2.35; 95% confidence interval [CI], 1.17–4.70; *p* = 0.02), to have lost weight (OR, 3.16; 95% CI, 1.40–7.09; *p* = 0.005), and to use a urinary catheter (OR, 3.33; 95% CI, 1.47–7.51; *p* = 0.004) (Table 2). The AUC of the DIVERT scale for predicting ED visits was 0.62 (95% CI: 0.52–0.72), that of the CARS was 0.59 (95% CI: 0.48–0.69), and that of the EARLI was 0.60 (95% CI: 0.50–0.70) (Figure 1).

**TABLE 2:** Relationships Between Patient Variables and ED Visits in Home Care Patients.

Variables	≧1 (vs. no) ED visits	
	Odds ratio (95% CI)	*p*‐Value
Age, years	1.02 (1.00, 1.05)	0.09
Gender, male	1.03 (0.52, 2.04)	0.94
DIVERT scale	1.57 (1.16, 2.11)	0.003
CARS	1.16 (1.00, 1.34)	0.047
EARLI	1.09 (1.02, 1.17)	0.02
Duration since the first visit, months	1.00 (1.00, 1.00)	0.28
Number of medications	0.99 (0.91, 1.07)	0.73
Prior ED visits within 6 months	3.14 (1.44, 6.84)	0.004
Receipt of ACP	2.35 (1.17, 4.70)	0.02
Presence of the caregiver	1.75 (0.58, 5.29)	0.32
Weight loss	3.16 (1.40, 7.09)	0.005
Use of a urinary catheter	3.33 (1.47, 7.51)	0.004
Medical history		
Urinary tract infection	2.81 (0.78, 10.1)	0.11
Heart failure	1.82 (0.89, 3.70)	0.10
COPD	0.92 (0.19, 4.35)	0.91
Renal failure	1.35 (0.42, 4.34)	0.62
Pneumonia	1.33 (0.27, 6.66)	0.73
Stroke	0.59 (0.26, 1.31)	0.19
Diabetes	0.93 (0.44, 1.96)	0.85
Coronary artery disease	2.41 (0.58, 10.06)	0.23

Abbreviations: ACP, advance care planning; CI, confidential interval; COPD, chronic obstructive pulmonary disease; ED, emergency department.

**FIGURE 2:**
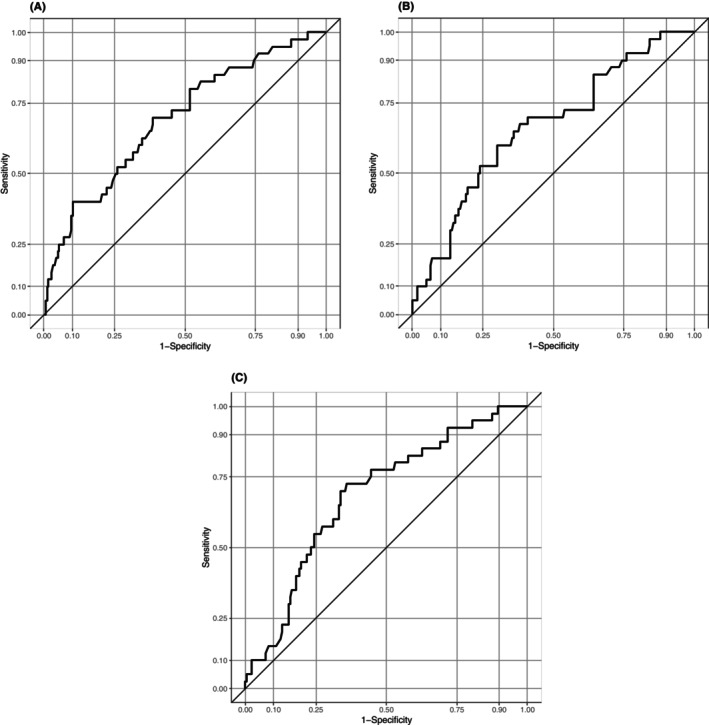
Receiver operating characteristic (ROC) curve analyses for each modified assessment scale: (A) the DIVERT scale, (B) the CARS, and (C) the EARLI. These ROC curves assessed the predictive performance of each modified assessment scale.

After the receipt of ACP and age were incorporated into each representative risk score in this study, the AUC of the DIVERT scale for predicting ED visits was 0.69 (95% CI: 0.60–0.78), and those of the CARS and the EARLI improved to 0.66 (95% CI: 0.57–0.76) and 0.68 (95% CI: 0.60–0.77), respectively (Figure 2).

**FIGURE 3:**
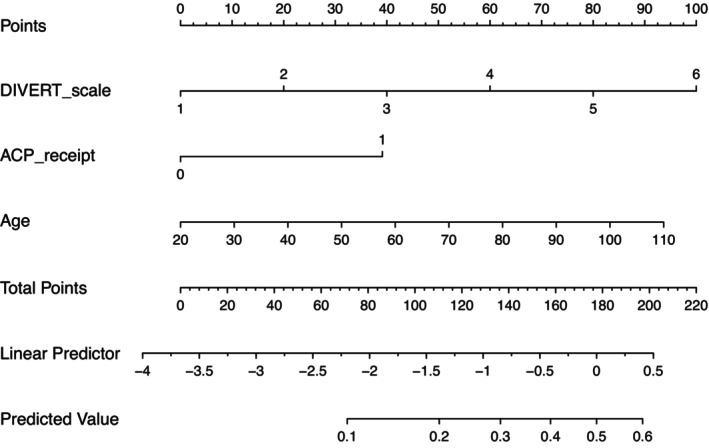
Nomogram for the modified DIVERT assessment scale for the prediction of emergency department visits by home health care patients in Japan. Points were assigned for the DIVERT scale, receipt of ACP, and age by drawing a line upward from each variable to the point. The sum of these three points became the total points. From that point, a vertical line was drawn downward, from which the linear predictor or predicted value was calculated. ACP, advance care planning.

We integrated ACP receipt and age into the DIVERT scale and constructed a nomogram for predicting ED visits over a 6 months period. The modified DIVERT scale assessment was used to estimate the probability of ED visits as shown in Figure 3.

## 4 DISCUSSION

To the best of our knowledge, this study is the first to validate the risk assessment scales for predicting ED visits among home health care patients in Japan. Among our sample of 224 patients, the AUC was 0.62, 0.59, and 0.60 for the DIVERT scale, the CARS, and the EARLI, respectively. The AUC for the DIVERT scale was superior to those of the CARS and the EARLI, and all AUCs were improved after receipt of ACP and age were integrated.

These scales have previously been validated in other countries: the AUC of the DIVERT scale was 0.52–0.72, CARS was 0.54–0.72, and EARLI was 0.54–0.70. Health care systems differ significantly between countries, and the results of these other measure validations might not be applicable in Japan. The AUC improvement observed in this study was relatively high compared with these values. ED visits affect the quality of life of home health care patients and their families. With developments and increased use of home health care in Japan, it has become critical to predict ED visits using risk assessment measures such as the DIVERT scale, the CARS, and the EARLI. Previous studies^9^, ^10^, ^11^, ^13^ that validated these scales in other countries calculated the receiver operating characteristics (ROC) curve. For the EARLI, all hospital admissions over a year were included. For the CARS, hospital admissions, or ED visits over a year were included.

Based on previous research, we hypothesized that receipt of ACP^15^, ^16^ and age^14^ could improve the prediction of ED visits. Therefore, these factors were integrated into the risk scales. The majority of ED visits are made by older individuals,^5^ and the likelihood of ED visits increases with age.^14^ Thus, this factor was thought likely to improve the accuracy of the scales. Receipt of ACP may avoid unnecessary ED visits.^15^, ^16^ However, previous studies have found that the risk of emergency transport does not change with ACP receipt.^21^, ^22^ We found higher rates of ACP receipt among patients who visited the ED than those who did not. This highlights the importance of ACP in the last stage of life.^23^, ^24^ Home health care physicians discuss ACP more frequently with patients with more severe health issues. Thus, our finding that those in receipt of ACP were more likely to visit the ED is likely because of poorer health among these patients.

This study validated the DIVERT scale, the CARS, and the EARLI in Japan. Adding age and receipt of ACP as variables on the scales improved their prediction of ED visits. This offers clinicians an improved ability to stratify the likelihood of ED visits for each patient and to provide preventive care for those at high risk. While a uniform preventive measure for all patients might be impossible, the findings of this study offer health policymakers increased opportunities to implement risk‐based preventive measures. In Canada, a randomized controlled trial that intervenes in patients based on risk is currently ongoing.^25^ Lastly, it is hoped that this study will provide a springboard for further multicenter studies offering more robust scale validation.

Our study had some potential limitations. First, there was the possibility of selection bias because of the focus on a single center. However, the majority of the patients were older, with a wide range of conditions. Second, we might not have been able to exclude confounding factors. Adjusting for confounding factors could lead to an overfitting of the model, which might compromise the generalizability of our findings, particularly in a dataset with this sample size. Therefore, we used univariate analysis to verify the improvement in accuracy provided by each factor. However, our analysis revealed that age and receipt of ACP had independent effects on the predictive accuracy of the risk assessment scales (DIVERT scale, CARS, and EARLI). We considered that including these variables as predictors is appropriate based on their effects, even when analyzed in a limited sample size. Therefore, we believe our findings to be broadly representative of Japanese home health care patients. Third, given the variability in the health care and emergency medical systems between countries, as well as differences in the practices of individual clinics, these results may not be directly comparable with those from other countries.

## 5 CONCLUSIONS

This study validated the risk assessment scales used to predict ED visits by home health care patients in Japan. The inclusion of receipt of ACP and age as variables improved the predictive accuracy of the scales, suggesting that modifying existing risk scales may improve the ability of home health care professionals to identify patients at higher risk of ED visits.

## FUNDING INFORMATION

This study was funded by the Yuumi Memorial Foundation.

## CONFLICT OF INTEREST STATEMENT

None declared.

## ETHICS STATEMENT

Ethics approval statement: This single‐center retrospective cohort study was approved by the Institutional Review Board of Fujita Health University Hospital and Midori Homon Clinic (approval number HM22‐368).

Patient consent statement: All study participants provided written informed consent and the study was conducted in accordance with the tenets of the 2013 revision of the Declaration of Helsinki.

Clinical trial registration: Not Applicable.

## Supporting information


Table S1.

## Data Availability

The datasets used and/or analyzed during the current study are available from the corresponding author on reasonable request.
