# Supplementary material for: Validating the DIVERT scales, CARS, and EARLI for predicting emergency department visits in home health care in Japan: A retrospective cohort study
Source: J Gen Fam Med. 2024 Nov 20;26(1):85–91. doi: 10.1002/jgf2.738 (PMC11702461; doi:10.1002/jgf2.738)
Supplement: Supplementary file 1 — Table S1. [file JGF2-26-85-s001.docx]

**Supplementary Table 1.** Variables and data sources used in the analysis

| Data Source | Variables |
| --- | --- |
| Codes used for health insurance | Age, sex, use of a urinary catheter,　duration since first visit, use of home oxygen therapy, terminal cancer status, medical history (urinary tract infection, heart failure, coronary artery disease, myocardial infarction, COPD, renal failure, pneumonia, stroke, diabetes, leg ulcer, cancer) |
| Clinic’s existing database | Death, socioeconomic status |
| Medical records | Prospects for functional improvement and number of medications |
| Patients and their caregivers | Prior ED visits within 6 months, presence of a caregiver, presence of symptoms (chest pain, dyspnea, dizziness, arrhythmia), mood symptoms, weight loss, any falls within 90 days, ability to go out without help, memory loss, self-assessment of health status, receipt of ACP |

Abbreviations: ACP, advanced care planning; ADL, activities of daily living; COPD, chronic obstructive pulmonary disease; ED, emergency department
